# Adherence to recommended practices for perioperative anesthesia care for older adults among US anesthesiologists: results from the ASA Committee on Geriatric Anesthesia-Perioperative Brain Health Initiative ASA member survey

**DOI:** 10.1186/s13741-020-0136-9

**Published:** 2020-02-25

**Authors:** Stacie Deiner, Lee A. Fleisher, Jacqueline M. Leung, Carol Peden, Thomas Miller, Mark D. Neuman, on behalf of the ASA Committee on Geriatric Anesthesia and the ASA Perioperative Brain Health Initiative, Mark D. Neuman, Shamsuddin Akhtar, Sona Arora, Itay Bentov, Sujatha Bhandary, Marek Brzezinski, Ruth E. Burstrom, Stacie Deiner, Kevin Evans, Jacqueline Leung, Michael C. Lewis, Sohail Mahboobi, Julie McSwain, Alec Rooke, Robert Whittington, Wendy Woo, Zhongcong Xie, Lee A. Fleisher, Stacie G. Deiner, Roderic G. Eckenhoff, Carol J. Peden, Alexander A. Hannenberg, Daniel J. Cole, James C. Eisenach, Hugh C. Hemmings, Keith A. Jones, Evan D. Kharasch, Jeffrey R. Kirsch, Aman Mahajan, Jeanine P. Wiener-Kronish

**Affiliations:** 1 Department of Anesthesiology, Perioperative and Pain Medicine, The Icahn School of Medicine at Mount Sinai, One Gustave Levy Place, New York, NY 10028 USA; 2 Department of Anesthesiology and Critical Care, University of Pennsylvania Perelman School of Medicine, Philadelphia, PA USA; 3 Penn Center for Perioperative Outcomes Research and Transformation, Philadelphia, PA USA; 4 Leonard Davis Institute of Health Economics, University of Pennsylvania, Philadelphia, PA USA; 5 Department of Anesthesia & Perioperative Care, University of California, San Francisco, CA USA; 6 Department of Anesthesiology, Keck School of Medicine, University of Southern California, Los Angeles, CA USA; 7 American Society of Anesthesiologists, Schaumberg, IL USA

**Keywords:** Geriatrics, Anesthesia, Cognition, Frailty

## Abstract

**Background:**

While specific practices for perioperative care of older adults have been recommended, little is known regarding adherence by US physician anesthesiologists to such practices. To address this gap in knowledge, the ASA Committee on Geriatric Anesthesia and the ASA Perioperative Brain Health Initiative undertook a survey of ASA members to characterize current practices related to perioperative care of older adults.

**Methods:**

We administered a web-based questionnaire with items assessing the proportion of practice focused on delivery of care to older adults, adherence to recommended practices for older surgical patients, resource needs to improve care, and practice characteristics.

**Results:**

Responses were collected between May 24, 2018, and June 29, 2018. A total of 25,587 ASA members were invited to participate, and 1737 answered at least one item (6.8%). 96.4% of respondents reported that they had cared for a patient aged 65 or older within the last year. 47.1% of respondents (95% confidence interval, 44.6%, 49.7%) reported using multimodal analgesia among patients aged 65 and older at least 90% of the time, and 25.5% (95% CI, 23.3%, 27.7%) provided preoperative information regarding postoperative cognitive changes at least 90% of the time. Over 80% of respondents reported that preoperative screening for frailty or dementia, postoperative screening for delirium, and preoperative geriatric consultation occurred in fewer than 10% of cases. Development of practice guidelines for geriatric anesthesia care and expansion of web-based resources were most frequently prioritized by respondents as initiatives to improve care in this domain.

**Discussion:**

Most survey respondents reported providing anesthesia care to older adults, but adherence to recommended practices varied across the six items assessed. Reported rates of screening for common geriatric syndromes, such as frailty, delirium, and dementia, were low among survey respondents. Respondents identified multiple opportunities for ASA initiatives to support efforts to improve care for older surgical patients.

## Background

The number of adults in the USA aged 65 and older will be more than double from 46 million today to more than 98 million by 2060, with the number of people ages 85 and older projected to be more than triple from 6 million to 20 million over the same period (Mather et al. 2015). These demographic changes carry major implications for the practice of anesthesiology. Adults over 65 comprise 15% of the US population but receive 35% of all inpatient surgery and 32% of all outpatient surgery performed in the USA each year; as a result, ensuring high-quality perioperative care for older adults now represents a core component of practice for many physician anesthesiologists (Hall et al. 2010; Cullen et al. 2009).

Older adults are at increased risk of complications and mortality after surgery (Turrentine et al. 2006; Monk et al. 2005). Beyond organ-based complications such as postoperative myocardial infarction, older adults may be more likely than other patients to experience specific syndromes, such as postoperative delirium, long-term cognitive changes, new dependence in activities of daily living (ADL), falls, and becoming pre-frail or frail (Turrentine et al. 2006; Stabenau et al. 2018). These syndromes may limit independence, increase the risk of mortality, and increase healthcare utilization (Mashour et al. 2015; Robinson et al. 2013).

In recognition of the distinct care needs of older surgical patients, major medical organizations in the USA and Europe have recently published guidelines and benchmarking efforts that have recommended specific practices for perioperative care of older adults (Chow et al. 2012; Mohanty et al. 2016; AGSEP on PD in OAE address:, Adults AGSEP on PD in O 2015; Roberts and Brox 2015; Aldecoa et al. 2017; Berian et al. 2018; Berger et al. 2018; Myles et al. 2007; White et al. 2019). Nonetheless, few data are presently available to characterize current practices among US physician anesthesiologists in this domain. From the standpoint of clinical care and health policy, such data are critical for establishing baseline measures for efforts to improve the quality of care and to inform selection of benchmarks for recommended practices by health systems, policy makers, and health care payers.

To address this gap in knowledge, the ASA Committee on Geriatric Anesthesia and the ASA Perioperative Brain Health Initiative undertook a survey in 2018 to characterize current practices related to perioperative care of older adults among physician anesthesiologist ASA members in active practice in the USA. In particular, the survey aimed to (1) characterize the proportion of cases reported by US physician anesthesiologists occurring among adults 65 or older; (2) describe adherence of US physician anesthesiologists to selected recommended practices for perioperative care of older adults, including practices related to the promotion of perioperative brain health; and (3) assess attitudes of US physician anesthesiologists regarding the value of selected types of resources or initiatives intended to support provision of high-quality perioperative care for older adults.

## Methods

### Questionnaire development

A web-based questionnaire was developed by members of the ASA Committee on Geriatric Anesthesia and the Perioperative Brain Health Initiative with input from the ASA Department of Analytics and Research. The final questionnaire contained 12 items across 3 domains (Appendix): the proportion of practice focused on delivery of anesthesia care to older adults (2 items), current provider or practice-level adherence to recommended practices among older surgical patients (6 items), resource needs to improve care for older adults (1 item), and practice characteristics (2 items). We selected 6 recommended practices for inclusion based on their presence within one or more relevant guidelines for care of surgical patients aged 65 and older: (1) preoperative frailty screening, (2) completion of a comprehensive preoperative geriatric evaluation, (3) use of multimodal analgesia, (4) preoperative cognitive screening, (5) postoperative screening for delirium, and (6) provision of information preoperatively regarding the risks of delirium or other cognitive disorder after surgery.

Adherence to recommended practices was assessed using a Likert-type response scale with the following levels: every time, usually (about 90% of the time), frequently (about 70% of the time), sometimes (about 50% of the time), occasionally (about 30% of the time), rarely (less than 10% of the time), and never (Matell and Jacoby 1971). Response options related to practice arrangement and primary work location used standard categories drawn from ASA member surveys and registration materials. Prior to distribution, the questionnaire was pilot tested for clarity among members of the ASA Committee on Geriatric Anesthesia and was approved for distribution by the Department of Analytics and Research by the ASA Executive Committee.

### Survey administration

The questionnaire was distributed by the Department of Analytics and Research to all US-based attending physician anesthesiologist ASA members in active practice via the SurveyMonkey web platform (SurveyMonkey, San Mateo, CA). Eligible ASA members received an initial e-mail with a unique web link to the online survey with an invitation message signed by leadership of the Committee on Geriatric Anesthesia and the Brain Health Initiative. Non-respondents received up to four reminder e-mails over a 6-week period. No incentives were provided for survey participation. This analysis was determined to be exempt from IRB review by the University of Pennsylvania IRB.

### Data analysis

We used descriptive statistics to characterize responses to individual survey items and calculated exact confidence intervals for all proportions. Where appropriate, response categories were collapsed to limit sparse cells.

As previous studies have demonstrated that most older surgical patients are treated in community hospitals rather than academic centers (Deiner et al. 2014), we conducted additional exploratory analyses to assess differences in adherence to recommended measures according to work setting and practice arrangement. Specifically, we used chi-squared tests to explore differences in the proportion of respondents indicating adherence at least 50% of the time among (1) respondents who reported their primary work location as a community hospital vs. respondents who reported working in other settings and (2) respondents who reported working in an academic group practice vs. respondents who reported working in other types of practice organizations. All analyses were carried out with Stata version 10.0 (Statacorp, College Station, TX)

## Results

Responses were collected between May 24, 2018, and June 29, 2018. A total of 25,587 ASA members received e-mails inviting them to participate, and 1737 answered at least one item (6.8%). For each item, the response rate varied between 6.8 and 5.8% (1737/25,587 and 1524/25,587).

Of the respondents, 65% were in a national, multispecialty, or single-specialty group, almost 30% were employed by an academic group, and 6% were in solo practice or another type of practice. Forty-nine percent reported working in a community hospital, 17% reported working in a medical school, and 15% reported working in a multilocation health system. Free-standing or office-based practitioners comprised 7% of the sample. Almost all respondents (96.4%) reported that they had cared for a patient aged 65 or older within the last year, and 60% responded that older adults comprised at least half of their cases.

Reported adherence to recommended practices varied across the items assessed (Table 1). Reported use of multimodal analgesia was high, with 47.1% of respondents indicating multimodal analgesia use among patients aged 65 and older at least 90% of the time. At the same time, 80.5% of respondents indicated screening for frailty in fewer than 10% of cases in adults aged 65 and older; 80.6% of respondents reported screening for signs of dementia or preexisting cognitive impairment in fewer than 10% of cases; 83.5% of anesthesiologists reported screening for delirium after surgery in fewer than 10% of cases; and over 80% reported that a preoperative evaluation by a geriatrician or geriatrics-trained provider occurred in fewer than 10% of cases. While 25.5% of practitioners did provide information on the risk of postoperative cognitive changes in at least 90% of cases, 30.5% discussed this information in 10% of cases or fewer.

**Table 1:** Responses to items assessing adherence to recommended practices (*N* = 1534)

	Completed “usually” or “every time” (90% or greater adherence): *n*, % (95% confidence interval)	Completed “frequently,” “sometimes,” or “occasionally” (30–70% adherence): *n*, % (95% confidence interval)	Completed “rarely” or “never” (less than 10% adherence): *n*, % (95% confidence interval)	Do not know
Preoperative frailty evaluation using a formal measurement scale	36, 2.3% (16.5%, 3.2%)	100, 6.5% (5.3%, 7.9%)	1235, 80.5% (78.4%, 82.5%)	163, 10.6% (9.1%, 12.3%)
Preoperative comprehensive geriatric evaluation	10, 0.7% (0.3%, 1.2%)	81, 5.3% (4.2%, 6.5%)	1262, 82.3% (80.3%, 84.1%)	181, 11.8% (10.2%, 13.5%)
Multimodal analgesia use in the first 24 hours after surgery	723, 47.1% (44.6%, 49.7%)	687, 44.8% (42.3%, 47.3%)	75, 4.9% (3.9%, 6.1%)	49, 3.2 % (2.4%, 4.2%)
Preoperative screening for dementia or other pre-existing cognitive impairment using a formal measurement scale	64, 4.2% (3.2%, 5.3%)	194, 12.6% (11.0%, 14.4%)	1267, 82.6% (80.6%, 84.5%)	N/A
Postoperative delirium screening using a formal measurement scale*	70, 4.6% (3.6%, 5.7%)	183, 11.9% (10.4%, 13.7%)	1280, 83.5% (81.5%, 85.3%)	N/A
Preoperative provision of information to patient or family regarding the risk of developing delirium or other cognitive disorders after surgery	391, 25.5% (23.3%, 27.7%)	675, 44.0 % (41.5%, 46.5%)	468, 30.5% (28.2%, 32.9%)	N/A

*Data available for 1533 respondents, 1 missing due to incomplete responses

These response patterns were qualitatively similar among respondents practicing at community hospitals vs. other practice locations (including government hospital, nongovernment hospital, medical school, free-standing surgery center, multilocation health system, office-based practice) and for respondents reporting employment by academic groups vs. others (Tables 2 and 3). At the same time, we did note some variability in guideline adherence according to primary work setting and practice arrangement. For example, compared with other respondents, those who identified their primary work setting as a community hospital less often indicated that at least 50% of cases among patients aged 65 or older underwent frailty screening (3.8% vs. 6.6%, *p* = 0.012), a preoperative geriatric evaluation (2.0% vs. 3.3%, *p* = 0.033), and postoperative delirium screening (8.9% vs. 14.2%, *p* = 0.001). In contrast, respondents who worked primarily at community hospitals (in comparison with government hospital, nongovernment hospital, medical school, free-standing surgery center, multilocation health system, office-based practice) more often reported discussing the risk of delirium and cognitive disorders with their patients before surgery (58.9% vs. 47.7%, *p* < 0.001). Compared with other respondents, those who reported employment by an academic group practice reported higher rates of frailty screening (9.5% vs. 3.4%, *p* < 0.001), a preoperative geriatric evaluation (5.9% vs. 1.7%, *p* < 0.001), preoperative dementia screening (16.9% vs. 8.8%, *p* < 0.001), and postoperative delirium screening (19.0% vs. 8.6%, *p* < 0.001). In contrast, respondents employed by academic practices less often reported discussing the risk of delirium and cognitive disorders before surgery (47.2% vs. 55.9%, *p* < 0.002).

**Table 2:** Percentages of respondents working in community hospitals vs. other settings reporting 50% or greater adherence to assessed care practices*

	Practice location: community hospital *n*/*N* (%)	Practice location: all other* *n*/*N* (%)	*p*
Preoperative frailty evaluation using a formal measurement scale	29/764 (3.8%)	51/717(6.6%)	0.012
Preoperative comprehensive geriatric evaluation	15/765 (2.0%)	29/767 (3.8%)	0.033
Multimodal analgesia use in the first 24 h after surgery	621/764 (81.3%)	651/768 (84.8%)	0.069
Preoperative screening for dementia or other pre-existing cognitive impairment using a formal measurement scale	75/764 (9.8%)	95/768 (12.4%)	0.112
Postoperative delirium screening using a formal measurement scale*	68/764 (8.9%)	109/767 (14.2%)	0.001
Preoperative provision of information to patient or family regarding the risk of developing delirium or other cognitive disorders after surgery	450/764 (58.9%)	366/768 (47.7%)	< 0.001

*Community hospital, government hospital, nongovernment hospital, medical school, free-standing surgery center, multilocation health system, office-based practice

**Table 3:** Percentages of respondents in academic vs. non-academic practice arrangements reporting 50% or greater adherence to assessed care practices

	Practice organization: academic practice n/N (%)	Practice organization: all other* n/N (%)	*p*
Preoperative frailty evaluation using a formal measurement scale	42/442 (9.5%)	37/1,084 (3.4%)	<0.001
Preoperative comprehensive geriatric evaluation	26/441 (5.9%)	18/1085 (1.7%)	<0.001
Multimodal analgesia use in the first 24 hours after surgery	386/442 (87.3%)	881/1084 (81.3%)	0.004
Preoperative screening for dementia or other pre-existing cognitive impairment using a formal measurement scale	75/443 (16.9%)	95/1,084 (8.8%)	<0.001
Postoperative delirium screening using a formal measurement scale*	84/442 (19.0%)	93/1,083 (8.6%)	<0.001
Preoperative provision of information to patient or family regarding the risk of developing delirium or other cognitive disorders after surgery	209/443 (47.2%)	605/1,083 (55.9%)	0.002

*Including solo practice/two-physician practice, group multispecialty practice, group national practice, group regional/multi-state practice, single-specialty group practice, and other

When asked to identify initiatives that should be prioritized by the ASA to improve perioperative anesthesia care for older adults, 79.5% identified development of relevant practice guidelines by the ASA, 67.5% identified web-based resources, and 50.3% identified assistance with implementing best practices at the local level through quality improvement or novel practice models. 12.7% of respondents identified development of a subspecialty training track in geriatric anesthesia or brain health as a priority for the ASA (Table 4).

**Table 4:** Responses to items assessing initiatives that should be prioritized by ASA to improve perioperative anesthesia care for older adults (*n* = 1524)

	Number selecting, % (95% confidence interval)
Development of practice guidelines in relevant areas	1212, 79.5% (77.4%, 81.5%)
Web-based educational resources	1029, 67.5% (65.1%, 69.9%)
Assistance with implementing best practices at the local level through quality improvement efforts or novel practice models such as the Perioperative Surgical Home	766, 50.3% (47.7%, 52.8%)
Additional educational sessions at the ASA Annual Meeting	695, 45.6% (43.1%, 48.1%)
Increased funding for related clinical, basic science, or health services research	547, 35.9% (33.5%, 38.4%)
Benchmarking efforts to provide feedback to ASA members on adherence to recommended practices	423, 27.8% (25.5%, 30.1%)
Development of a formal subspecialist training track in geriatric anesthesia or brain health	193, 12.7% (11.0%, 14.4%)
Other	86, 5.6% (4.5%, 6.9%)

## Discussion

Among 1737 US-based physician anesthesiologist ASA members responding to a web-based survey, nearly all respondents indicated that they provided surgical anesthesia care to adults aged 65 and older in the course of their practice, with a majority indicating that such patients constituted 50% or more of their cases. While respondents commonly reported routine use of multimodal analgesia among older surgical patients, reported adherence to other recommended practices was less common. Among patients over 65 who were treated in their practice over the prior 12 months, most respondents reported low rates of preoperative geriatric evaluations, preoperative screening for frailty or cognitive disorders, and postoperative delirium screening. One quarter of respondents indicated routinely discussing the risk of postoperative delirium or other cognitive disorders.

Beyond the above findings, we identified several potential initiatives that respondents indicated could aid in efforts to improve geriatric anesthesia care, including development of dedicated practice guidelines for geriatric anesthesia, and expansion of web-based resources to guide practice. Finally, we identified variation in adherence to recommended practices across respondents based on reported primary work locations and reported group structure. For example, respondents employed by academic groups reported relatively greater adherence to screening recommendations for geriatric syndromes than other respondents; respondents employed by nonacademic groups and those who worked primarily in community hospitals reported more often presenting patients with information prior to surgery on the risk of delirium or other neurocognitive disorders. Despite these differences, the absolute adherence to all assessed practices other than use of multimodal analgesia was reported to be very low regardless of the primary work setting or employing group structure.

The findings of the present study should be interpreted with caution. The overall response rate was 6.8%; while this falls within the range of response rates achieved by other ASA member surveys, it implies a substantial amount of missing data relevant to the characterization of patterns of care by the ASA membership. There is a wide literature regarding survey recruitment strategy including method of delivery, ensuring acknowledgment from potential respondents to ensure the survey was received, and use of incentives (Phillips et al. 2016). In retrospect, addition of an acknowledgment for the e-mail survey would be a useful low-barrier way to increase participation. While we note a low rate of reported adherence to many of the practices assessed, it is possible that the present survey may actually overestimate adherence compared with ASA members in general for two reasons. First, as participation in this survey is voluntary, it is possible that those individuals who chose to respond to our survey may have been more likely to have an interest in anesthesia for older adults, and thus comprised a group who would be more likely to be aware of, and potentially adhere to, recommendations assessed here. Second, due to social desirability bias or “faking good,” reported adherence to recommended practices by survey respondents may have been greater than actual adherence in practice. While our analysis of differences in reported care patterns according to work location and practice arrangement may help to stimulate hypotheses for future research, it is important that these analyses also be interpreted with caution due to the low overall response rate of the present survey. In particular, since we are unable to rule out important differences between respondents and non-respondents across the practice arrangements and work locations assessed here, these findings cannot support definitive conclusions regarding differences in care based on practice arrangement or primary work location. Finally, since we did not collect any data from US physician anesthesiologists who were not ASA members at the time of the survey, we cannot comment on the generalizability of this work to non-member physicians.

Guidelines for the care of older surgical patients exist in many countries. The European Society has published Delirium Guidelines, and the Association of Anesthetists of Great Britain and Ireland has published Guidelines for Perioperative Care of the Elderly(White et al. 2019; Aldecoa et al. 2017). Very high-quality research regarding delirium and postoperative cognitive dysfunction has been performed and published in Australia (Evered and Silbert 2018; Evered et al. 2011). There has been an increase in research in the area of brain health in Asia, specifically China, and South America (Su et al. 2016; Avelino-Silva et al. 2014). Much less information is available from Africa, which has overall a lower life expectancy (United Nations 2014).

Despite these limitations, the present study offers an important starting point for efforts to improve care and outcomes for older adults undergoing surgery and anesthesia. Our finding of low overall rates of screening for geriatric syndromes in the perioperative period highlights potential opportunities to improve adherence to recommended practices over time. As such, these findings highlight a need for further work to understand potential promoters and barriers to adherence to recommended screening practices across diverse settings and to characterize potential strategies that may increase adherence. Implementation of practice changes in other areas such as stroke care and back pain faces challenges which may be relevant to the Brain Health Initiative in surgical patients such as need for institutional support, increased awareness, limited skill or confidence with new techniques, and persistence with popular but not recommended techniques (Baatiema et al. 2017; Slade et al. 2016). At the same time, the observations of our respondents raise important questions regarding the feasibility of implementing several of the recommended practices here across diverse clinical settings and highlight a need for further work to determine the optimal role of physician anesthesiologists vs. other clinicians in carrying out aspects of recommended care for older adults before, during, and after surgery. Indeed, it is worth noting that most recommendations assessed here are drawn from guidelines developed by professional societies outside of anesthesia and as such may not consider important feasibility issues that may limit adherence to such recommendations in practice. Development of specialty-specific guidelines by the ASA represents an initiative that was highly prioritized by respondents to the survey and may serve as an opportunity to evaluate the recommendations assessed here in terms of their feasibility within current anesthesia practice.

In conclusion, while we observed that survey respondents reported frequently providing anesthesia care to older adults in practice, adherence to recommended practices varied markedly across the items assessed, and we identified multiple areas in which opportunities may exist to improve such adherence. In particular, reported rates of pre- and postoperative screening for common geriatric syndromes, such as frailty, delirium, and dementia, were low among survey respondents. Respondents identified multiple opportunities for ASA initiatives to support efforts to improve care for older surgical patients, including potential development of guidelines and provision of additional educational or practice management resources.

## Data Availability

**C**onsult with T. Miller and M. Neuman

## Appendix

Questionnaire items

1. Over the last 12 months, did you provide surgical anesthesia care for patients aged 65 or older?
  Response options: Yes; No
2. Please choose the statement below that BEST characterizes the proportion of those patients for whom you provided surgical anesthesia over the last 12 months who were 65 or OLDER.
  Response options: All or nearly all of my cases (90% or more); Most of my cases (about 70%); about half of my cases (about 50%); Some of my cases (about 30%); A few of my cases (about 10%)

*Among patients aged 65 and older for whom you provided surgical anesthesia over the last 12 months…*

3. …how often was a PREOPERATIVE FRAILTY EVALUATION performed using a formal measurement scale, such as the Frailty Phenotype, Frailty Index, Modified Frailty Index, either by an anesthesiologist or another type of care provider (e.g., geriatrician, internist)?
  Response options: Every time; Usually (90% or more); Frequently (about 70%); Sometimes (about 50%); Occasionally (about 30%); Rarely (about 10%); Never
4. …how often was a PREOPERATIVE GERIATRIC EVALUATION performed by a care provider specializing in geriatric medicine (e.g., geriatrician, geriatric nurse practitioner)?
  Response options: Every time; Usually (90% or more); Frequently (about 70%); Sometimes (about 50%); Occasionally (about 30%); Rarely (about 10%); Never
5. ...how often was MULTIMODAL ANALGESIA, defined as use of more than one modality for pain treatment, used in the first 24 hours after surgery?
  Response options: Every time; Usually (90% or more); Frequently (about 70%); Sometimes (about 50%); Occasionally (about 30%); Rarely (about 10%); Never
6. …how often did you specifically assess for signs of dementia or other pre-existing cognitive impairment preoperatively using a CLINICAL ASSESSMENT TOOL, such as the Montreal Cognitive Assessment (MOCA), the Mini-Mental Status Examination (MMSE) or Mini-Cog?
  Response options: Every time; Usually (90% or more); Frequently (about 70%); Sometimes (about 50%) Occasionally (about 30%); Rarely (about 10%); Never
7. …how often did you specifically assess for signs of DELIRIUM preoperatively or post-operatively using a CLINICAL ASSESSMENT TOOL, such as the Confusion Assessment Method (CAM) or the Delirium Rating Scale (DRS)?
  Response options: Every time; Usually (90% or more); Frequently (about 70%); Sometimes (about 50%); Occasionally (about 30%); Rarely (about 10%); Never
8. …how often did you provide the patient or their family with specific information prior to surgery on their risk of developing delirium or other cognitive disorders after surgery?
  Response options: Every time; Usually (90% or more); Frequently (about 70%); Sometimes (about 50%); Occasionally (about 30%); Rarely (about 10%); Never
9. The following is a list of potential initiatives that the ASA Committee on Geriatric Anesthesia and the ASA Brain Health Initiative could consider developing for ASA members. Please check all that you believe should be prioritized:

- Development of practice guidelines in relevant areas
- Additional educational sessions at the ASA Annual Meeting
- Web-based educational resources
- Development of a formal subspecialist training track in geriatric anesthesia or brain health
- Increased funding for related clinical, basic science, or health services research
- Benchmarking efforts to provide feedback to ASA members on adherence to recommended practices
- Assistance with implementing best practices at the local level through quality improvement efforts or novel practice models such as the Perioperative Surgical Home
- Other (please specify)

10. How would you describe the organization of your clinical practice?
  Response options: Solo; Group: multispecialty; Group: national; Group: academic; Group: single specialty; Other
11. What is your primary clinical work setting?
  Response options: Community hospital; Government hospital; Nongovernment hospital; Medical school; Free-standing surgery center; Multi-location health system; Office based practice

## Abbreviations

ASA: American Society of Anesthesiology
IRB: Institution Review Board
