# Correction: Adherence to recommended practices for perioperative anesthesia care for older adults among US anesthesiologists: results from the ASA Committee on Geriatric Anesthesia-Perioperative Brain Health Initiative ASA member survey

**DOI:** 10.1186/s13741-025-00612-x

**Published:** 2025-12-03

**Authors:** Stacie Deiner, Lee A. Fleisher, Jacqueline M. Leung, Carol Peden, Thomas Miller, Mark D. Neuman

**Affiliations:** 1 Department of Anesthesiology, Perioperative and Pain Medicine, The Icahn School of Medicine at Mount Sinai, One Gustave Levy Place, New York, NY 10028 USA; 2 Department of Anesthesiology and Critical Care, University of Pennsylvania Perelman School of Medicine, Philadelphia, PA USA; 3 Penn Center for Perioperative Outcomes Research and Transformation, Philadelphia, PA USA; 4 Leonard Davis Institute of Health Economics, University of Pennsylvania, Philadelphia, PA USA; 5 Department of Anesthesia & Perioperative Care, University of California, San Francisco, CA USA; 6 Department of Anesthesiology, Keck School of Medicine, University of Southern California, Los Angeles, CA USA; 7 American Society of Anesthesiologists, Schaumberg, IL USA

**Correction: Perioper Med 9, 6 (2020)**

**https://doi.org/10.1186/s13741-020-0136-9**

Following the publication of the original article (Deiner et al. 2020), the authors identified an error in the Competing interests section. The statement “Lee Fleisher is the Editor-in-Chief of the Perioperative Medicine” should be added to the section. The section should read:

**Competing interests**

Dr. Peden is a shareholder in Fitzroy Health and has received consultancy fees from Merck. Dr. Deiner has received consultancy fees from Merck, product support from Covidien, and received payment as a legal expert witness. Lee Fleisher is the Editor-in-Chief of the Perioperative Medicine. The remaining authors have no conflicts of interest to disclose.

The Competing interests section has been updated above and the original article (Deiner et al. 2020) has been corrected.
